# 

**Published:** 1910-06-04

**Authors:** 


**Pneumonia.**

Beware of excessive feeding by enthusiastic nurses; let the patient have plenty of fluids—we do not use tea enough in the treatment of acute disease; it is useful as a mild stimulant.—*Dr. J. W. Carr.*

**Trade Dermatitis.**

Supposing it is impracticable for the patient to change his occupation, the management of a case resolves itself into avoiding the deprivation of the skin of the lubricant, which naturally protects it from irritation and supplying one where deficient. At night the patient should wash his hands with oil and then with soap and water, well rinsing in several changes of water. During the night ointment may be applied which is removed in the morning with dry wool. The parts may be rubbed at intervals during the day with a salve-stick of wax and lanoline. Washing should be limited to once daily.—*Dr. Norman Walker.*

### THE GRADUATES' MEDICAL SCHOOLS.

DIARY FOR THE WEEK, JUNE 6 to JUNE 11.

**ST. MARK'S HOSPITAL FOR DISEASES OF THE RECTUM,** City Road, E.C.

At 2.30 p.m.—**June 8**, Mr. Swinford Edwards, **Fistula.**

**LONDON SCHOOL OF CLINICAL MEDICINE,** Seamen's Hospital, Greenwich, S.E.

At 2.15 p.m.—**June 7**, Prof. Hewlett, **Prophylactic Vaccination.**
4 p.m.—**June 9**, Mr. Pendlebury, **Surgical Treatment of Empyema and Hydrocephalus in Children.**

**NATIONAL HOSPITAL FOR THE PARALYSED AND EPILEPTIC,** Queen Square, Bloomsbury, W.C.

At 3.30 p.m.—**June 7**, Mr. Percy Sargent, **Nerve Anastomosis.**
3.30 p.m.—**June 10**, Dr. Aldren Turner, **Treatment of Epilepsy.**

**CENTRAL LONDON THROAT AND EAR HOSPITAL,** Gray's Inn Road, W.C.

At 3.45 p.m.—**June 7** and **10**, Mr. Stuart Low, **The Accessory Sinuses.**

**THE BROMPTON HOSPITAL FOR CONSUMPTION AND DISEASES OF THE CHEST,** Fulham Road, S.W.

At 4 p.m.—**June 8**, Dr. Perkins, **Angina Pectoris.**

**NORTH-EAST LONDON POST-GRADUATE COLLEGE,** Prince of Wales's General Hospital, Tottenham, N.

At 4.30 p.m.—**June 7**, Mr. J. H. Evans, **Demonstration of Selected Surgical Cases.**
4.30 p.m.—**June 10**, Mr. R. P. Brooks, **The Surgical Treatment of High Myopia.**

**LONDON HOMŒOPATHIC HOSPITAL,** Great Ormond Street, W.C.

At 5 p.m.—**June 10**, Dr. J. G. Blackley, **Some Acute Skin Cases.**

**MEDICAL GRADUATES' COLLEGE AND POLYCLINIC,** 22 Chenies Street, W.C.

At 4 p.m.—**June 6**, Dr. James Galloway, **Clinique (skin).**
5.15 p.m.—**June 6**, Dr. G. E. Herman, **Post-partum Hæmorrhage.**
4 p.m.—**June 7**, Dr. Guthrie Rankin, **Clinique (medical).**
5.15 p.m.—**June 7**, Dr. James Taylor, **Some Points in the Treatment of Nervous Diseases.**
4 p.m.—**June 8**, Mr. Cecil H. Leaf, **Clinique (surgical).**
5.15 p.m.—**June 8**, Dr. A. E. Giles, **Pregnancy after Abdominal Operations.**
4 p.m.—**June 9**, Dr. F. J. Poynton, **Clinique (medical).**
5.15 p.m.—**June 9**, Mr. M. P. Mayo Collier, **On Deformities of the Face and Upper and Lower Jaws, due to Nasal Obstruction.**
4 p.m.—**June 10**, Mr. Ernest Clarke, **Clinique (eye).**

**ROYAL SOCIETY OF MEDICINE,** 15 Cavendish Square, W. (Temporary address during building.)

At 5.30 p.m.—**June 7, Surgical Section :**
In the Large Theatre, Medical School, University College, W.C.
*Discussion* on "The present position of the treatment of syphilis." (To be continued at the next meeting on June 14.) The discussion will be opened by Mr. Ernest Lane, and continued by Sir Jonathan Hutchinson, Dr. Alexander Fleming, Mr. Arthur Shillitoe, Mr. H. W. Bayly, Mr. J. E. R. McDonagh, Dr. F. J. Smith, Mr. C. F. Marshall, Dr. G. Pernet, Mr. Charles Gibbs, and Captain Rennie.

At 5 p.m.—**June 8, Special Meeting of Fellows of the Society.**
At Morley Hall, George Street, Hanover Square, W.
*Discussion* on "Vaccine Therapy: its administration, value and limitations" (fourth meeting). Re-opened by Dr. Arthur Latham, and continued by Mr. Maynard Smith, Dr. Alexander Fleming, Dr. J. T. Horder, Dr. Carmalt-Jones, and Dr. Rufenacht Walters.

At 7.45 p.m.—**June 9, Obstetrical and Gynæcological Section:**
*Specimens* : Dr. Drummond Maxwell : Cystic degeneration of mole in Fallopian tube. Dr. Inglis Parsons : (1) Fibroma of Fallopian tube; (2) Fibrotic uterus causing persistent menorrhagia.
*Paper* : Sir William Japp Sinclair : "The prophylaxis and treatment of the slighter ailments resulting from puerperal sepsis."

At 5 p.m.—**June 10, General Meeting of Fellows:**
Election of candidates for Fellowship.
—— p.m.—**Clinical Section:**
*Annual General Meeting* : Election of Officers and Council.
*Cases* : Dr. R. G. Hebb : Lymphadenoma. Mr. R. C. Elmslie : (1) Three cases of Schatter's disease; (2) Fracture of the humerus at the site of an innocent cyst. Dr. H. D. Rolleston : Jaundice with areas of unpigmented skin. Dr. Poynton and Mr. Wilfred Trotter : Cardiolysis. Mr. L. Vernon Cargill : Acromegaly with tumour of the pituitary body. Mr. Lockhart Mummery : (?)Hyperplastic tuberculosis of the pelvic colon. Mr. Cyril Nitch : Congenital hairy mole. Mr. Wilfred Harris : Trigeminal neuralgia treated by alcohol injections.

---

Mr. Thomas Windsor, of Brownslow, Great Budworth, Cheshire, M.R.C.S., L.S.A., who died on April 13 at the age of seventy-eight, left estate valued at £38,162 gross, and £25,498 net. He left bequests of medical and other books and MSS. to the library of the Surgeon-General at Washington (D.C.), U.S.A., the Manchester Medical Society, Owens College Library, and the Ryland Library; and £100 to the Mauldeth Hospital. Subject to these and other provisions he left the rest of his property to his executors to use "for the relief of suffering in such way and at such time as may appear best to them, with these restrictions, that nothing shall be given to any hospital or dispensary, or medical society, or medical institution; nor shall any sum be given to any church or charity subject to any particular religious body or sect." It appears that the amount ultimately available for this trust will be about £25,000.

A council meeting of the Association of Medical Officers of Health was held in London on May 25, 1910, at 3 p.m. Dr. Crookshank presided over a good attendance. Dr. Belilios (Hon. Secretary) reported the accession of new members, and Dr. Gibbs-Smith (Hon. Treasurer) a satisfactory increase in subscriptions and balance. A letter was read from the General Secretary of the British Medical Association giving consent for a general meeting to be held in London during the approaching annual meeting of the British Medical Association. It was agreed to arrange for the meeting to be held on Friday, July 29, and to be followed by a dinner. Provisions of the draft Public Health Officers Bill of the British Medical Association were considered, and it was resolved to take steps to oppose in Parliament any Bill differentiating between part-time and whole-time medical officers of health in respect of condition of tenure. It was decided to issue a memorandum stating the position of affairs at the present time, and defining the policy of the Association of Medical Officers of Health.

It is only fair that those hospital workers who go about, according to Nature, on four feet instead of on two should have their work and their fellows' acknowledgment of their work duly mentioned. We are therefore glad to say that "Bruce," whose importunate beggings at Swindon and elsewhere on behalf of the hospitals are well known to everyone who has stopped at that station, took part again this year in the Hospital Sunday Parade at Reading, wearing all his decorations. The chief of these is the silver collar the dog received from Miss Ackerley, the late matron of the Victoria Hospital, Swindon, as an acknowledgment of his ability and energy in raising £100 for her hospital. The actual fastening of the collar was "performed" by the Mayor of Swindon, while Colonel Calley, the division's member of Parliament, fixed a shield bearing medals presented by the districts to which "Bruce" had travelled in the course of his philanthropic work. His example is genuine enough to be worth recording, and we congratulate the dog (and Mr. Beal, who trained him) on his services as a worker in the hospital field. These services represent nearly a solid £200 in cash, and the work is said to have involved 2,500 miles of walking. What man could do more?

### The Hospital World.

With all classes of the King's subjects, remarks the *British Temperance Advocate*, we desire to associate ourselves in the universal tribute which has been tendered to the memory of King Edward, and to express our sense of the nation's loss through the untoward event which shocked us all with its dramatic suddenness on May 6. We have lost a great King who strove for the welfare of all his people, and for peace amongst the nations of the earth. In our own immediate movement we have to thank him amongst other evidences of sympathy for : (1) His support of temperance work in the Army and Navy. One of the last acts of his life was to send from Sandringham on May 2 a telegram of congratulation to Miss Agnes Weston on her birthday, whilst his favour to the Army Temperance Association was shown by his express permission that it should be called "The Royal." (2) His personal deliverance that his health drunk in water was as heartily appreciated as when drunk in wine or other intoxicants. (3) Many years ago he cleared the Sandringham estate of all liquor licenses, an individual option working out into prohibition, which some day, we hope, will be in the hands of subjects of the King.

### Appointments.

Edwin Ash, M.D. Lond., pathologist to the London Temperance Hospital, has been appointed assistant physician to the Italian Hospital, London.

Mr. Joseph H. Bennett, L.R.C.P., has been appointed assistant house surgeon, and Mr. Allan Semple, M.B., Ch.B. (Glas.), assistant house physician to the Leicester Infirmary as from June 1.

The following district medical officers have been re-appointed for one year:—Salford district, Mr. C. H. Bradbury, M.R.C.S., L.R.C.P. (Lond.); Broughton district, Dr. A. C. Clarke, M.D.; Pendleton district, Mr. J. Lithgow Davie, M.B., Ch.B.

Dr. E. P. Minett has been appointed assistant Government bacteriologist at the Public Hospital, Georgetown, British Guiana. Dr. Minett was educated at Guy's Hospital, where he worked for two years in the Opsonic and Vaccine Department, and was assistant bacteriologist. Subsequently he was appointed bacteriologist to the Moorfields Hospital, and more recently still has been carrying on special research at the Cancer Hospital on the value of vaccines in the treatment of malignant disease.

### GIFTS AND BEQUESTS.

The trustees of Smith's Charity have sent £270 to Queen Charlotte's Lying-in Hospital.

Lady Stewart, of Chilworth Manor, near Guildford, has left £100 to the Winchester Hospital.

The Royal Dental Hospital, Leicester Square, has received a legacy of £1,000 by the will of the late Mrs. Elizabeth Cooper, per John H. Hale, Esq., the sole executor.

The treasurer of the Middlesex Hospital has received from the trustees of the Smith (Kensington Estate) Charity a grant of £536.

The New Hospital for Women has received £270 from the trustees of Smith's Charity.

The treasurer of the Hampstead General Hospital has received a grant of £536 from the trustees of the Smith (Kensington Estate) Charity.

Mr. John Thomas Gay, of Stockton, Wilts, farmer, has left £500 to the Governors of the Salisbury Infirmary and £250 to the Wylye Valley Nursing Association for the benefit of the parish of Stockton, the income only of the fund to be so applied, except in the case of a serious epidemic in the district, when the trustees have power to expend not more than £50 out of capital to cope with it.

Among the donations, running into three figures, in response to the special appeal for University College Hospital, are: "Well Wisher," £300; the Hon. F. H. Baring, £100; Messrs. N. M. Rothschild and Sons, per H. Lucas, Esq., £100; the Worshipful Company of Grocers, £100; Miss Emma Goldsmid, £50; Miss Isabel Goldsmid, £50; E. Filliter, Esq., £50.

The Dreadnought Hospital, Greenwich, has received 2,000 taels (£251 2s. 4d.) from the Imperial Chinese Government in recognition of services rendered to sick and injured Chinese sailors.

Mr. Henry Rothwell Daglish, of New Romney, Kent, farmer, has left £2,000 to Guy's Hospital, London.

The Metropolitan Hospital Sunday Fund has passed resolutions of sympathy with King George and the Queen Mother, which were proposed by the Archdeacon of London.

Mrs. Helen Crowther, of Rochdale, has bequeathed £3,000 to Rochdale Infirmary, £4,000 to Rochdale District Nursing Association, £1,000 to the Pendlebury General Hospital and Dispensary for Sick Children, the above legacies to be associated with the name of her late husband, in memory of whom she bequeathed the same.

Sir Julius Wernher, Bart., has given £1,000 to endow a bed in response to the special appeal for £10,000 before June 1 on behalf of University College Hospital, to which Mr. Herbert Tilley, F.R.C.S., has also contributed £1,000 for a similar purpose.

### JOTTINGS.

South Pole Expedition.—Messrs. Robert Boyle and Son, ventilating engineers, 64 Holborn Viaduct, have supplied the ventilators for the iron huts to be used in connection with Captain Scott's expedition to the South Pole.

Mr. William Herring has promised £1,000 towards the Hospital Sunday Fund, in the hope that the collections may be doubled this year, as an act of special devotion to his late Majesty King Edward, whose interest in the hospitals our readers know full well.

The West London Hospital, Hammersmith, says a letter in the *Times*, has just received a postal order for 2s. 6d. from a bricklayer's labourer, "on the anniversary of my operation," which is quite reminiscent of Pepys, who gave a dinner to his friends "on the anniversary of the day when I was cut for stone."

A characteristic instance of King Edward's practical kindness and interest in hospitals was his gift of an operation table to the Devonshire Hospital at Buxton in 1905. King Edward and Queen Alexandra visited that institution, and his Majesty, seeing in the course of his inspection that a table was wanted in the theatre, sent a modern one as a present, which Lord Derby aptly recalled at the recent annual meeting.

The Invalid Children's Aid Association is appealing for £300 to continue, on the larger scale a house would make possible, its work of boarding out tuberculous children in country homes under the supervision of a doctor and a lady visitor. The sum needed is £500, to which the Duchess of Marlborough has given £200, on condition that the remainder is subscribed promptly. The success of the experiment so far makes this occasion of extending it a chance not to be lost.

### TO HOSPITAL SECRETARIES AND OTHERS.

We invite contributions from any of our readers, and shall be glad to pay, according to length, for items of news for the institutional section (including appointments, resignations, gifts, etc.) which we may publish. All payments for manuscripts used are made as early as possible after the beginning of each quarter.

### COMING EVENTS OF THE WEEK.

Monday, June 6.—Opening St. Vincent's Cripples Home, Clarence Road, Clapham Park, S.W. (by the Lord Mayor), 3.30 p.m.

Tuesday, June 7.—Lady Salisbury opens sale of work in aid of Royal Hospital for Incurables, Putney, 2.30 p.m.

Wednesday, June 8.—Harben Lecture by Sir W. Leishmann, Institute of Public Health, 6 p.m.

Sale of Work, Royal Hospital for Incurables, Putney.

Annual Meeting, St. John's Hospital for Diseases of the Skin at Leicester Square, W.C., 4 p.m.

League of Mercy, Poplar and Limehouse District: Concert given by Mr. and Mrs. Godding at the Limehouse Church Institute at 8 p.m.

Thursday, June 9.—Annual General Meeting Convalescent Homes Association, 32 Sackville Street, 4.30 p.m.

Sale of Work, Royal Hospital for Incurables, Putney.

Mr. Sargent's demonstration at Queen Square Hospital for Paralysed and Epileptic, 5 p.m.

League of Mercy, East Marylebone District: The Hon. Mrs. George Lawson Johnston, "At Home," 29 Portman Square, 4 p.m.

Friday, June 10.—Research Defence Society's Annual Meeting, Royal College of Physicians, Pall Mall, 5 p.m.

Dr. Noel Bardswell, medical superintendent of King Edward VII. Sanatorium, is about to publish, through Messrs. A. and C. Black, a book entitled "Advice to Consumptives: Home Treatment and After-Care." The object of the book is to give practical advice, more especially to the general public, as to how best to combat consumption and other forms of tuberculous disease. A chapter is devoted to an account of the nature of tuberculosis and the way in which the disease may be cured. The *rationale* of the modern treatment of tuberculosis is also explained. Other chapters deal with fresh air, food, rest, exercise, recreations, clothing, baths, and personal hygiene generally. The question of the best occupation is also discussed, and some consideration is given to the pros and cons of emigration and of marriage.

